# Effects of Implementing the Short-Term Assessment of Risk and Treatability for Mechanical Restraint in a Forensic Male Population: A Stepped-Wedge, Cluster-Randomized Design

**DOI:** 10.3389/fpsyt.2022.822295

**Published:** 2022-02-24

**Authors:** Jacob Hvidhjelm, Mette Brandt-Christensen, Christian Delcomyn, Jette Møllerhøj, Volkert Siersma, Jesper Bak

**Affiliations:** ^1^Clinical Mental Health and Nursing Research Unit, Mental Health Center Sct Hans, Copenhagen University Hospital – Mental Health Services CPH, Copenhagen, Denmark; ^2^Mental Health Centre Sct Hans, Copenhagen University Hospital – Mental Health Services CPH, Copenhagen, Denmark; ^3^Head of Centre, Competence Centre for Forensic Psychiatry, Mental Health Centre Sct Hans, Copenhagen University Hospital – Mental Health Services CPH, Copenhagen, Denmark; ^4^The Research Unit for General Practice and Section of General Practice, Department of Public Health, University of Copenhagen, Copenhagen, Denmark

**Keywords:** mental health, psychiatry, forensic, coercion, mechanical restraint, risk assessment, Short-Term Assessment of Risk and Treatability

## Abstract

The assessment and formulation of the risk of violence and other unwanted behaviors at forensic psychiatric facilities have been attempted for decades. Structured professional judgment tools, such as the Short-Term Assessment of Risk and Treatability (START), are among the recent attempts to overcome the challenge of accomplishing these goals. This study examined the effect of implementing START in clinical practice for the most serious adverse events among the target group of severely mentally ill forensic psychiatric inpatients. Results were based on the use of mechanical restraints as an outcome. This study is a pragmatic, stepped-wedge, cluster-randomized controlled trial and was conducted over 5 years. It included eight forensic psychiatric units. Fifty out of 156 patients who had a basic aggression score of more than 0 were included in the study. We found that the rate of mechanical restraint use within the START period were 82% [relative risk (RR) = 0.18], lower than those outside of the START period. Patients evaluated within the START period were also found to have a 36% (RR = 0.64) lower risk of having higher Brøset Violence Checklist scores than patients evaluated outside the START period. Previous studies on START have primarily focused on validation, the predictive capability of the assessment, and implementation. We were only able to identify one study that aimed to identify the benefits and outcomes of START in a forensic setting. This study showed a significant reduction in the chance for inpatients in a forensic psychiatric facility to become mechanically restrained during periods where the START was used as risk assessment.

## Introduction

Valid and reliable measures to assess the risk of violence and other challenging behaviors at forensic psychiatric facilities have been in demand for decades, and several structured professional judgment tools have been developed and introduced into clinical practice (1–8). The Short-Term Assessment of Risk and Treatability (START), a 20-item structured professional judgment instrument designed for recurrent clinical assessments within inpatient and community contexts, is among the most recently developed assessment tools (9). The predictive validity of START for several problem behaviors is generally considered good to excellent within a short to moderate timeframe (10–14). The implementation of START in clinical practice to focus on the patient's strengths and vulnerabilities has been assumed to provide enhanced opportunities to predict and prevent severe violence and self-harm. Scientific reports of using START alone or comparing START with other risk assessment tools in secure mental health settings have been published (11, 15–18). Previous research has primarily focused on its validation and predictive ability (19, 20). The most recent study focused on summarizing item values as a single concept, primarily for research purposes (21). To our knowledge, no previous study has highlighted the reduction in mechanical restraint use as an outcome. Since 2010, there has been an increased focus among Danish politicians and health authorities on reducing the use of coercion in hospital psychiatric departments. In 2014, the Danish government ordered a reduction of mechanical restraint use in inpatient settings by half before 2020, signaling that this was considered the most intrusive type of coercion applied. Additionally, it was a political goal that all types of coercion should decrease during this period. Considerable efforts and resources have been applied, leading to a reduction in the use of coercion; however, the overall goal of reducing the use of mechanical restraints by 50% compared to baseline (years 2011–2013) was not fully achieved by the end of 2020 (22). The present study was initiated by implementing START in clinical practice between May 1, 2012, and April 30, 2017, at a large, medium-secure, forensic mental health facility in the Capital Region of Denmark. All forensic psychiatric facilities in Denmark are publicly funded and are subject to public health authorities. This study aimed to examine the effects of START implementation in clinical practice on the most serious adverse events as expressed by the necessity for mechanical restraint use among the target group of severely mentally ill forensic psychiatric inpatients.

## Materials and Methods

### Study Design

Randomized controlled trials are considered the gold standard when evaluating the effectiveness of interventions in the healthcare context. However, randomized controlled trials have some weaknesses in a workplace setting. Therefore, to overcome these challenges, we decided to apply a stepped-wedge, cluster-randomized design, which has some advantages over classic randomized controlled trials in this setting (23, 24). We attempted to overcome some of the difficulties associated with the use of the stepped-wedge design. During our study, it was possible to gradually implement the intervention in all participating units and motivate the staff and patients.

Another gold standard is following the intention-to-treat principle. This is not preferable in a naturalistic scenario, such as ours, because it is not always possible to rescreen the patients within the maximum effect period of START (which we determined to be 6 months). Furthermore, because some units lost key staff members, they could not evaluate patients with START until new key staff members were trained. Therefore, if we had followed the intention-to-treat principle, we would not have been able to evaluate the effect of START; instead, we would have obtained the effect of the ability of the unit to perform START.

This study used the definition of mechanical restraint as defined by Bowers et al. (25) (the use of restraining straps, belts, or other equipment to restrict movement). This definition refers only to the restraint of inpatients at psychiatric hospitals. The following conditions must be present to legally initiate mechanical restraint according to the Danish Mental Health Act (as translated by the authors):

“Mechanical restraint may be used only when necessary to prevent patients from the following: (1) Exposing their body or health or the body or health of others to danger. (2) Pursuing or in any other way grossly molesting fellow patients. (3) Committing significant acts of vandalism” (26).

According to the law in Denmark, all coercive episodes must be reported to the national database for coercion (26).

### Other Instruments

#### Staff Observation Aggression Scale-Revised

All aggressive or violent incidents were systematically recorded using the electronic version of the Staff Observation Aggression Scale-Revised (SOAS-R). The SOAS-R is an instrument that reports damaging or threatening aggressive behaviors toward an object and/or humans. The SOAS-R is completed each time a staff member witnesses aggressive or violent behavior by a patient. The SOAS-R has been tested and validated by several studies (27–29). With the SOAS-R scoring system, the severity of an incidence can be rated from 0 to 22 points; a score >8 is considered severe. The SOAS-R has shown good inter-rater reliability, with kappa values of 0.61–0.74 (27). The SOAS-R has been used in daily clinical practice since 2008. Staff is trained to register a SOAS-R whenever they witness or are themselves exposed to a violent incident. All registrations are entered in an IT system (designed by Frenzs B.V., Nijmegen, the Netherlands).

#### Brøset Violence Checklist

The Brøset Violence Checklist (BVC) is used to evaluate the presence (score of 1) or absence (score of 0) of six symptoms: confusion, irritability, boisterousness, physical threats, verbal threats, and attacks on objects. According to standard guidelines (30), a total score of 0 (none of these behaviors present) suggests that the risk of violence is low. A score of 1–2 suggests that the risk is moderate and preventive measures should be taken. A score of 3 or more suggests that the risk of violence is high, immediate preventive measures are required, and plans for managing an attack should be activated (30, 31). The BVC was implemented in 2005 and recorded in patients files on daily basis.

### Population and Timeframe

This study was conducted over 5 years, from May 1, 2012, to April 30, 2017, at the Forensic Department of the Mental Health Centre Sct Hans, Mental Health Services, in the Capital Region of Denmark. Ten units comprise the Forensic Department; however, one unit was excluded because it had served as a pilot unit, and one was excluded because it did not use mechanical restraints. The mean number of beds per unit was 9.4 (range, 8–10 beds).

We included all male forensic patients who displayed one or more basic aggressive episodes. A basic aggressive episode is defined as an episode involving a total SOAS-R score of more than 8 during the first month of inclusion in the study. A total of 50 male patients were included. The reason for excluding patients without one or more basic aggressive episodes was associated with applying mechanical restraints. In Denmark, mechanical restraints are only initiated if the patients are aggressive (toward themselves, others, or things). Therefore, if implementing the START would reduce the use of mechanical restraints, then it would only be possible to detect if the patients had aggression issues. To select patients with aggression issues, we selected those who experienced one or more severe aggressive episodes during the first month of inclusion in the study based on the assumption that those would be the ones most at risk for requiring mechanical restraints.

All units admitted both male and female patients. A total of 13 female patients were admitted during the study period (only three with a basic aggressive episode score were in the included eight units). However, they were excluded because they were presumed to have different associations between START and mechanical restraint use compared to the male patients and because they comprised a sample too small for separate analysis.

### Sampling and Data Collection

**Figure 1:**
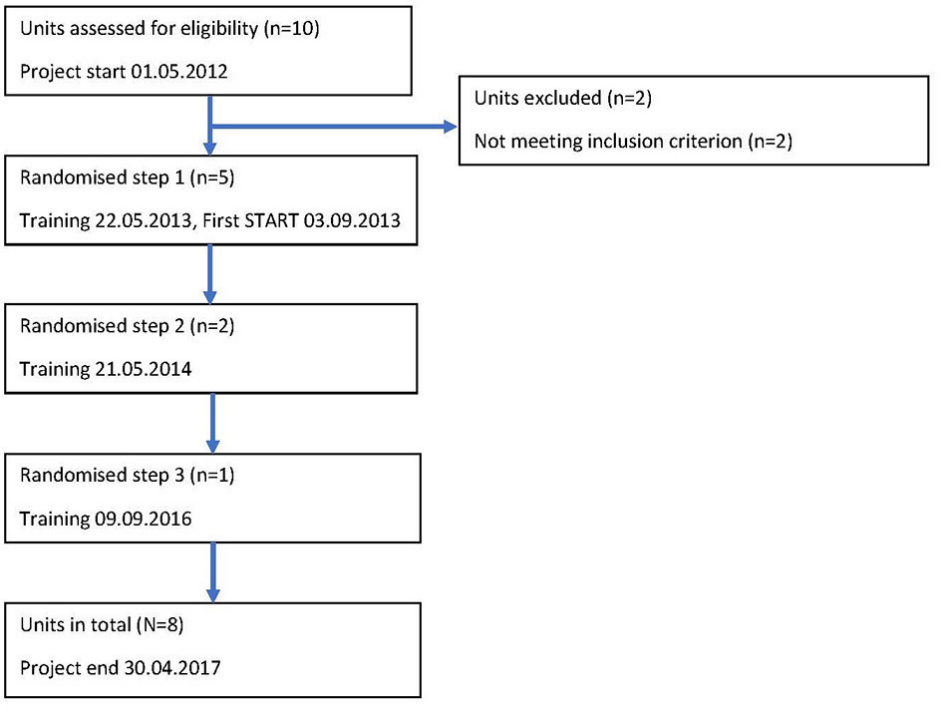
Flowchart for the stepped wedge inclusion of units.

Of the 10 units of the Forensic Department, eight were used for the study. Five units were randomized to step one: beginning the training for key staff to teach them how to screen patients using START on May 22, 2013. Two units were randomized to step two: beginning the training for key staff 1 year later, on May 21, 2014. The last unit began the training for key staff 2.5 years after step two had begun, on September 9, 2016. All data were retrospectively gathered in May 2017 (see Figure 1). The included units were randomized by one of the researchers (JB), using the random number generator in the statistics software that was used.

Key staff members (nurses) were trained in leading the START assessment meeting with multidisiplanery staff attending (e.g., nurses, assistant nurses, psychologists, and psychiatrist). The initial START assessment is often more time consuming than the follow-up assessments and therefore there was a natural decrease in the time spent doing a START assessment—from 1.5 h down to 30 min.

**Figure 2:**
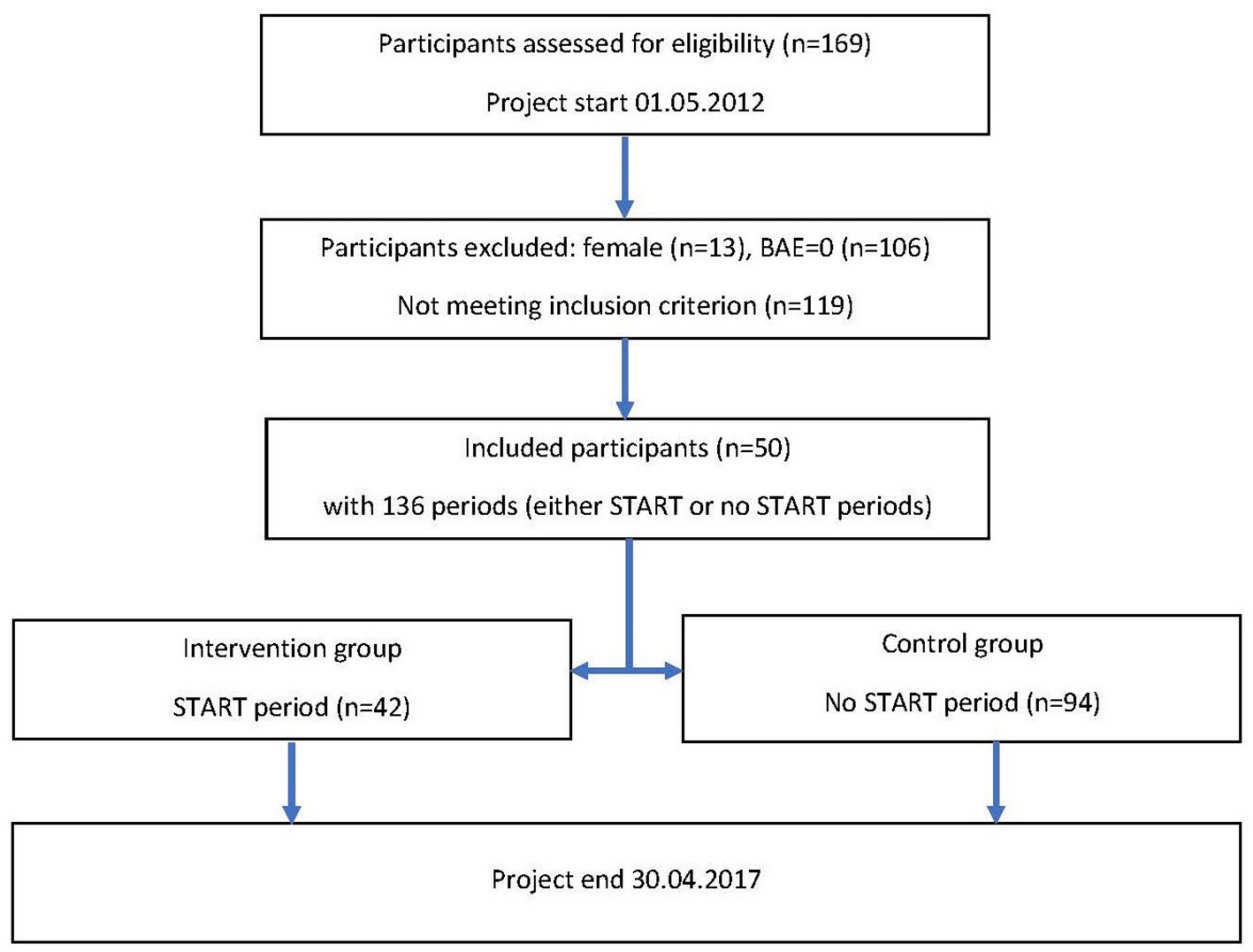
Flowchart of participants.

The vast majority of patients admitted to a forensic unit is admitted under court order. In Denmark, forensic psychiatry is part of general psychiatry and not a specialty in itself. The overall responsibility for initiating and implementing treatment is placed upon the treating psychiatrist and always happens in collaboration with other staff. A total of 239 patients were admitted during the study period between May 1, 2012, and April 30, 2017. After the first process of excluding patients from both the pilot unit, and the unit that did not use coercive measures, we were left with 169 patients who were assessed for eligibility. Based on the argumentation above, 13 female patients were additionally excluded. The remaining 156 patients were then rated based on their Basic Aggressive Episodes (Basic Aggressive Episode “BAE”: episode involving a SOAS-R score >8 during the first month of participation in the study). A total of 50 (36%) patients who displayed one or more BAE were included in the study. The START period began when the patients underwent the first START evaluation, and it proceeded until the patients did not undergo the START evaluation for 6 months. After that time, they began the control period; however, another START period could have begun if another START evaluation had been performed. This procedure resulted in 42 START periods and 92 control periods for these patients (see Figure 2).

### Outcome Variables

The primary outcome was the occurrence of mechanical restraint use. Secondary outcomes were the total duration (in minutes) of mechanical restraint use, total coercive episodes (number of physical restraint episodes, episodes of acute forced medication, episodes of one to one observation (without patient consent) and mechanical restraint episodes) and the number of BVC scores more than 2 (a score of 3–6 indicated a severe risk of violence within the next 24 h). The BVC scores were determined three times every 24 h. In a Danish context, mechanical restraint is considered the most severe type of coercion, as the use of seclusion rooms is not allowed. Therefore, mechanical restraint was selected as the outcome measure.

We selected mechanical restraint *(coercion)* rather than SOAS-R as our primary outcome measure. The SOAS-R outcome variable is known to have a relative high degree of underreporting (32). As mentioned earlier registration is mandatory by law and therefore we assume much less underreporting on mechanical restraint than on the SOAS-R.

### Potential Confounders

Normally, randomization eliminates the effect of potential confounding variables caused by even distribution. To ensure this in our study, we gathered information about the most important potential confounding variables for this group of patients: age, diagnosis, length of hospitalization before study inclusion, and psychoactive substance use.

#### Previous Study Findings

According to previous studies, younger men required mechanical restraint use more often, and patients diagnosed with schizophrenia or schizotypal and delusional disorders (WHO ICD-10-codes F20-F29) required mechanical restraint use more often (33). Patients required mechanical restraint use more often at the beginning of their hospitalization period (33, 34). Further, patients diagnosed with mental and behavioral disorders because of psychoactive substance (WHO ICD-10-codes F10-F19) use experienced mechanical restraint use more often (33).

### Data Analysis

Negative binomial regression was performed to assess the incidence of mechanical restraint use when START evaluations were performed compared to when no START evaluations were performed (35). To assess a cluster effect of “unit”, we calculated the proportion of the explained variance attributable to a “unit” as the difference of the coefficients of determination (*R*^2^) of a model with and without “unit” included as categorical variable, divided by the *R*^2^ of a model with “unit” included. The most important covariates were tested for differences in the BAE groups, the START group, and the control group using the chi-squared test. However, because there were both paired and unpaired data, the Cochran-Mantel-Haenszel test was also performed. We analyzed the patient-level data to eliminate the cluster effect. Statistical significance was set at *p* < 0.05. All analyses were performed using SPSS Statistics for Windows version 25.0 (36).

### Approval and Ethics

Interventions such as those included in the present study do not require approval from the scientific ethical committee system in Denmark, because it does not include any drugs or biological material and the intervention is regarded part of the natural improvement of care and treatment. The Danish Data Protection Agency (RHP-2013-002, I-Suite no. 02053) approved this study. We received permission from the Center Management and Clinical Management of the Forensic Department to perform this study. We also received permission from the developers of START to use their method during this study (April 1, 2014). This study followed the ethical guidelines for nursing research in the Nordic countries (37) and the recommendations on the legal protection of persons suffering from mental disorders, especially those placed as involuntary patients (38).

## Results

Patients who were 28 to 35 years of age had a higher prevalence of having one or more basic aggressive episodes at the beginning of the study period (BAE > 0, 22.0% vs. BAE = 0, 32.1%), but the difference was not significant (*p* = 0.20). Additionally, patients with one or more basic aggressive episodes at the beginning of the study period (basic aggressive episodes >0) had a higher prevalence of schizophrenia, schizotypal or delusional disorder, and other delusional disorders (F20-F29) (BAE > 0, 84.0% vs. BAE = 0, 75.5%), but the difference was not significant (*p* = 0.23) (Table 1).

**Table 1:** Descriptive statistics of background variables of the whole population.

	**BAE** **=** **0 (*****n*** **=** **106)**		**BAE** **>** **0 (*****n*** **=** **50)**		**Total (*****N*** **=** **156)**		**χ** ^**2**^
	***n***	**%**	***n***	**%**	***N***	**%**	***p*-value**
Age when the patient was included in the study							
<27 years	28	26.4%	16	32.0%	44	28.2%	0.47
28–35 years	34	32.1%	11	22.0%	45	28.8%	0.20
36–45 years	25	23.6%	13	26.0%	38	24.4%	0.74
>45 years	19	17.9%	10	20.0%	29	18.6%	0.76
Diagnoses of schizophrenia, schizotypal and delusional disorders (F20–F29)	80	75.5%	42	84.0%	122	78.2%	0.23
Length of hospitalization before the patient were included in the study (<1 year)	55	51.9%	29	58.0%	84	53.8%	0.48
Diagnoses of psychoactive substance use, primary or secondary (F10–19)	68	64.2%	32	64.0%	100	64.1%	0.99

*BAE (Basic Aggressive Episodes) = the sum of SOAS-R scorings above 8, the first month the patient were in the study. Patients with at least one BAE (BAE > 0) was included in the study. χ^2^, Chi square test for the difference (Δ) between the BAE = 0 group and the BAE > 0 group*.

A total of 296 (72.7%) START assessments (on both patients with a BAE score = 0 and BAE above 0) out of 407 was preformed during the study period with a mean of 4.1 month between assessments. One patient had 17 START assessments preformed during the study period.

Patients in the intervention group (START period) had been hospitalized before being included in the study (*p* = 0.01) (Table 2). The Cochran-Mantel-Haenszel test results were similar for these patients.

**Table 2:** Descriptive statistics of the background variables of the START group and the control group.

	**START group (*****n*** **=** **42)**		**Control group (*****n*** **=** **94)**		**Total (*****N*** **=** **136)**		**χ** ^**2**^
	***n***	**%**	***n***	**%**	***n***	**%**	***p*-value**
Age when the patient was included in the study							
<27 years	11	26.2%	26	27.7%	37	27.2%	0.86
28–35 years	9	21.4%	19	20.2%	28	20.6%	0.87
36–45 years	12	28.6%	28	29.8%	40	29.4%	0.89
>45 years	10	23.8%	21	22.3%	31	22.8%	0.85
Diagnoses of schizophrenia, schizotypal and delusional disorders (F20–F29)	39	92.9%	82	87.2%	121	89.0%	0.33
Length of hospitalization before the patient were included in the study (<1 year)	13	31.0%	51	54.3%	64	47.1%	0.01^*^
Diagnoses of psychoactive substance use, primary or secondary (F10–19)	25	59.5%	57	60.6%	82	60.3%	0.53

*A START period is the day and time of the START rating plus 6 months. A control period is all other periods where the patient is hospitalized. The 50 patients (BAE > 0) had a total of 136 periods and all patients in the START group also was a part of the control group. 22 patients only participated in the control group with 29 periods. χ^2^, Chi square test for the difference between the START group and the control group*.

* *p ≤ 0.05*.

The rate of mechanical restraint use within the START period was 82% lower than that outside the START period [relative risk (RR) = 0.18; 95% confidence interval (CI), 0.08–0.41; *p* < 0.01; *p* = 3.01 × 10–5]. The results of an adjusted analysis (RR = 0.17; 95% CI, 0.08–0.37; *p* < 0.01; *p* = 6.0 × 10–6) were similar, indicating that incidence differences could not be explained by confounding factors. The proportion of fit attributable to “unit” was 0.04 (4%) (Table 3).

**Table 3:** Effect of START on the outcome variables: mechanical restraint use, total coercion and BVC episodes.

	**START group** **(*****n*** **=** **42)**		**Control group** **(*****n*** **=** **94)**		**Unadjusted analysis**^**1**^ **(*****N*** **=** **136)**				**Adjusted analysis**^**2**^ **(*****N*** **=** **136)**				**Proportion of fit attributable to “unit”** ^**3**^
	**Rate (#/month)**		**Rate (#/month)**		**RR**	**95% Wald confidence interval of RR**		***p*** **-value**	**RR**	**95% Wald confidence interval of RR**		***p*** **-value**	
	**Mean**	**SD**	**Mean**	**SD**		**Lower**	**Upper**			**Lower**	**Upper**		
MR^4^ episodes	0.03	0.10	0.18	0.39	0.18	0.08	0.41	0.00^**^	0.17	0.08	0.37	0.00^**^	0.04
Duration in minutes of MR^4^ episodes	274	1652	609	1950	0.01	0.00	0.03	0.00^**^	0.00	0.00	0.01	0.00^**^	0.01
Total coercive episodes^5^	0.12	0.32	0.39	0.94	0.37	0.19	0.74	0.01^*^	0.33	0.19	0.60	0.00^**^	0.01
Number of BVC^6^ episodes (>2)	.96	1.38	1.42	1.72	0.64	0.44	0.91	0.01^*^	0.60	0.43	0.86	0.01^**^	0.08

1 *Negative Binominal Regression, Offset = log. to the length of the period, Repeated Subject = Patient ID, Adjusted for Units (cluster effect)*.

2 *Further adjusted for: Age, Diagnoses, Length of hospitalization, and Psychoactive substance use*.

3 *Proportion of fit attributable to “unit” = the proportion of R2 with and without units (clusters)*.

4 *Mechanical Restraint*.

5 *Total coercive episodes = number of physical restraint episodes, episodes of acute forced medication, episodes of one to one observation (without patient consent) and mechanical restraint episodes*.

6 *BVC, Brøset Violence Checklist*.

* *p ≤ 0.05*.

** *p ≤ 0.01*.

The duration of mechanical restraint use was 99% lower within the START period than outside the START period (RR = 0.01; 95% CI, 0.00–0.01; *p* < 0.01; *p* = 2.0 × 10–14). The results of an adjusted analysis (RR = 0.002; 95% CI, 0.001–0.006; *p* < 0.01; *p* = 0.0 × 10-E) were similar, indicating that the incidence difference could not be explained by confounding factors. The proportion of fit attributable to “unit” was 0.01 (1%). The very small RR, could probably be explained by a few patients, mechanical restrained for a long period of time. Therefor, the analyses of duration should not be the primary result (Table 3).

The rate of total use of coercion within the START period was 63% lower than that outside the START period [relative risk (RR) = 0.37; 95% confidence interval (CI), 0.19–0.74; *p* < 0.01]. The results of an adjusted analysis (RR = 0.33; 95% CI, 0.19–0.60; *p* < 0.00) were similar, indicating that incidence differences could not be explained by confounding factors. These results (RR = 0.37 compared to RR = 0.18), indicates that some of the mechanical restraint episodes is converted to a lesser intrusive kind of coercion (which in it self would be a positive result), but not to a degree that impacts the results especially. The proportion of fit attributable to “unit” was 0.01 (1%) (Table 3).

Finally, the risk of having a BVC score more than 0 (BVC score >2 indicated a severe risk of violence within the next 24 h) within the START period was 36% lower than that outside the START period (RR = 0.64; 95% CI, 0.44–0.91; *p* = 0.01). The results of an adjusted analysis (RR = 0.60; 95% CI, 0.43–0.86; *p* = 0.01) were similar, indicating that the incidence difference could not be explained by confounding. The proportion of fit attributable to “unit” was 0.08 (8%) (Table 3).

## Discussion

The stepped-wedge design was chosen for both ethical and practical reasons. Ethical considerations included the absence of equipoise because there is evidence that START will do more good than harm. Therefore, it would be unethical to withhold its implementation from participants. Practically, the design solved the problem of simultaneous implementation in half the units and many logistical and practical problems (39, 40). Additionally, the outcomes were available from routinely collected data (BVC and SOAS-R) (40). The design also allowed us to include a large number of patients. The clustering of sites confined to one geographical site and the number of sites could have been limitations to the generalizability of the current study (35, 39, 41).

In an ideal world, where the time between START assessments had been possible to keep as recommended in the manual (9), and where key employees that was responsible for arranging START assessments meetings was not ill or had left their position in the department, and that sufficient resources were available so that the team performing the assessment had opportunity to meet. We would have expected to have collected 407 START assessments but collected 296 assessments. It might not even be possible to reach a complesion rate of 73% in this ideal world, as we were able to reach in our naturalistic scenario.

Previous research has primarily focused on the validation and predictive ability of START. Few studies have focused on its implementation and outcomes, especially in inpatient settings. Kroppan et al. (11) described START implementation in two phases at Forensic Mental Services at Brøset in Trondheim, Norway, during a 10-year period. Their study showed increased interdisciplinary participation with the implementation of START. The research group also highlighted that the implementation of START requires continuous efforts. The application of the assessments to the treatment plans proved challenging when the study was performed, although progression from the assessment to the assessment-treatment phase during the implementation period was identified. In the current study, the START implementation was performed by clinicians who had experience using START in the clinical setting and experience training clinicians to use START. In each unit, two superusers were educated about START and were in charge of its implementation in their unit. They were supervised by the superusers of the pilot unit, who were supervised by teachers from Norway. The superusers met during the implementation phase to rate cases together. The intensive use of external supervision and continuous follow-up could explain the positive implementation process and significant study results.

To our knowledge, the study by Gunenc et al. (17) is the only one that focused on the benefits and outcomes of START in a forensic inpatient setting. They expected to find a reduction in adverse behaviors (physical and verbal aggression, self-harm, victimization, self-neglect, unauthorized leave, and substance abuse); however, they found no significant changes in physical or verbal aggression over time. There was no reduction in self-harm or substance abuse incidents during the 3 months after the START evaluation. Despite the power calculations, the authors (17) indicated that the sample size (*n* = 50) was one explanation for their results. During our study, we opted for a study period that was considerably longer than the 3-month comparison period before and after the assessment follow-up period used by Gunenc et al. (17). The longer study period might be one of the reasons for our significant results. We only included patients who demonstrated that they can use aggression to express themselves; therefore, patients were included if they had a BAE score >0.

According to the research literature within the field of forensic psychiatry and personal recovery processes, START is emphasized as an important risk assessment tool because it focuses on the resources, strengths, and protective factors in addition to the weaknesses and risks of the individual patients (42, 43). Managing risk as well as positive risk-taking and protective factors are key offender recovery elements in specialized forensic services, and this implies the involvement of mentally disordered offenders in their risk assessment and management and reduction of specific risks (44–46). Consequently, it would be relevant to develop a patient version of START to support and increase the involvement of the mentally disordered offenders, thereby supporting the processes of personal recovery. Lockertsen et al. (47) added items to the original version of the BVC and studied their extended version; for example, their Self-Report Risk Scale provided patients with an opportunity to predict their risk of violence. As a result, their study showed that expressing one's risk resulted in better short-term accuracy of predicting violence than the original BVC (45).

In a systematic review by Goulet et al. (48) its being concluded that with implementation of a program that focues on reducing seclusion and reduction it is possible to affected the use of such methodes in a positive way. The review defines such programs as programs including the following key-components; Leadership, training, post-seclusion and restraint review, patient involvement, use of prevention tools and forcus on the therapeutic environment. The use of START is one component in one of the mentioned six key-components, namely “Prevention tools”. In our study the only implemented or used component that in daily practice separates the intervention group and the control group is the use of START.

A patient version of START would probably help to increase patient awareness of risk factors and highlight the responsibilities of the patients and the professionals working with them. As pointed out in the substantial literature about recovery processes in forensic settings, such approaches can help patients regain a sense of control over their lives, thereby providing hope (39). Furthermore, the implementation of START in specialized forensic outpatient services would be an interesting area for future research in the context of Denmark. Troquete et al. (49) examined the preventive effect of combining START and a shared care protocol in forensic outpatient settings without finding a significant preventive effect on recidivism to violent or criminal behavior. They (49) stated that the proportion of clients in the intervention group not receiving the intervention or receiving it only once was a limitation. Additionally, they did not have much success motivating the case managers to perform activities during their study, which was a limitation to their study (49). Therefore, this topic requires further scrutiny.

## Data Availability Statement

The raw data supporting the conclusions of this article will be made available by the authors, without undue reservation.

## Ethics Statement

Written informed consent was not obtained from the individual(s) for the publication of any potentially identifiable images or data included in this article.

## Author Contributions

JH, CD, and JB carried out the project. VS supervised the analysis. JH and JB took lead in writing the manuscript. All authors discussed the results and contributed to the final manuscript. All authors contributed to the article and approved the submitted version.

## Conflict of Interest

The authors declare that the research was conducted in the absence of any commercial or financial relationships that could be construed as a potential conflict of interest.

## Publisher's Note

All claims expressed in this article are solely those of the authors and do not necessarily represent those of their affiliated organizations, or those of the publisher, the editors and the reviewers. Any product that may be evaluated in this article, or claim that may be made by its manufacturer, is not guaranteed or endorsed by the publisher.
